# Supplementary material for: Complexome profiling of the Chlamydomonas psb28 mutant reveals TEF5 as an early PSII assembly factor
Source: Plant Cell. 2025 Mar 17;37(6):koaf055. doi: 10.1093/plcell/koaf055 (PMC12164590; doi:10.1093/plcell/koaf055)
Supplement: koaf055_Supplementary_Data [file koaf055_supplementary_data.zip › psb28_interactive.zip!/plots/intensity/qualityPlots/peptides/CGL21_Cre09.g402950.t1.1.html]

### Cre09.g402950.t1.1

Trivial name: CGL21  
SeqCoverage [median]: 30.30  
Mass: 47.20 kDa  
  
 

| Sequence | MissedCleavage | Mass [Da] | wtIntensity | muIntensity | Score |
| --- | --- | --- | --- | --- | --- |
| AEREEVVSAGGEK | 1 | 1359.66 | 1734.46 | 4237.03 | 104.17 |
| AGLTPTELK | 0 | 928.52 | 415.64 | 1247.31 | 86.10 |
| AGLTPTELKR | 1 | 1084.62 | 3300.73 | 8120.69 | 91.55 |
| ASGFTAVEVFR | 0 | 1182.60 | 0.00 | 3702.34 | 81.34 |
| ELVDGLNK | 0 | 886.48 | 304.60 | 3662.62 | 85.27 |
| FLLAGNR | 0 | 789.45 | 419.45 | 4499.10 | 86.34 |
| KFDQGAVEDLVALK | 1 | 1531.82 | 0.00 | 7066.69 | 98.58 |
| LVGQGAESEGGAGNVADIGK | 0 | 1827.90 | 382.01 | 3524.72 | 126.77 |
| NTQSFLTTTLPGK | 0 | 1406.74 | 0.00 | 2379.40 | 103.22 |
| TGLGLTDGDAGEALR | 0 | 1444.72 | 0.00 | 6209.20 | 88.44 |
| TGLGLTDGDAGEALRER | 1 | 1729.86 | 478.39 | 10646.38 | 87.02 |
| VYDKYGTLMLNTEGLTLSGAQR | 1 | 2429.23 | 0.00 | 3741.88 | 69.72 |
|  |  |  | 7035.3 | 59037.4 |  |
